# Pericarditis due to *Campylobacter coli* infection: a case report

**DOI:** 10.1186/s12879-023-08293-x

**Published:** 2023-05-10

**Authors:** Shohei Katsuno, Chieko Itamoto, Isano Hase

**Affiliations:** 1 Department of Pharmacy, Nagano Chuo Hospital, Nagano City, Japan; 2 Department of Cardiology, Nagano Chuo Hospital, Nagano City, Japan; 3 Department of Pulmonology, Nagano Chuo Hospital, 1570, Nishitsuruga-machi, Nagano City, Nagano Japan

**Keywords:** *Campylobacter coli*, Pericarditis

## Abstract

*Campylobacter* spp. is a gram-negative bacillus that causes infectious enteritis and consists of several species, including *Campylobacter jejuni*, *Campylobacter coli*, and *Campylobacter fetus*. Although *C. jejuni* and *C. coli* cause infectious enteritis primarily in immunocompetent hosts, *C. fetus* causes extraintestinal infections such as septicemia, meningitis, and perinatal infections in immunocompromised hosts, as well as myopericarditis in rare cases. Only a few cases of infectious myo(peri)carditis associated with *C. coli* in immunocompetent hosts have been reported. These studies concentrated on antecedent *C. coli* enterocolitis and never demonstrated a positive culture in the pericardial fluid.

A 72-year-old Japanese man presented with a 2-week fever, cough, and vomiting lasting. He was on hemodialysis for polycystic kidney disease, as well as medication for diabetes and hypertension. A chest computed tomography (CT) scan and a transthoracic echocardiogram revealed bilateral pleural fluid and large pericardial fluid at the time of admission. *C. coli* was identified from blood culture samples and blood-tinged pericardial fluid. He was successfully treated with antibacterial chemotherapy as well as pericardial fluid drainage and was discharged from the hospital with no complications.

In this case, the presence of *C. coli* in the pericardial fluid confirmed the diagnosis of *C. coli* pericarditis. *C. coli* may cause septic pericarditis in immunocompromised hosts, despite typically causing only enteritis.

## Background

The bacteria *Campylobacter* spp. is well known as the causative agent of infectious enteritis. As of December 2014, *Campylobacter* had 26 species, two provisional species, and nine subspecies, including *Campylobacter jejuni*, *Campylobacter coli*, and *Campylobacter fetus* [1]. These organisms’ properties differ depending on their species. *C. jejuni* and *C. coli* cause primarily infectious enteritis primarily in immunocompetent hosts [1]. Although *C. fetus* rarely causes infectious enteritis, it frequently causes extraintestinal infections in immunocompromised hosts, including septicemia, meningitis, and perinatal infections [2–4]. *C. fetus* was the most frequently identified species in *Campylobacter*-associated bacteremia (94 [53%] of 178 patients) [5], whereas *C. coli* was only found infrequently (16 [9%] of 178 patients) [5]. *C. coli* is less prevalent than *C. jejuni and C. fetus*. There are few reports on *C. coli*-caused infectious diseases other than infectious enteritis.

Pericarditis is a pericardial inflammatory syndrome. Pericarditis and myocarditis have similar aetiologies, and overlapping forms are common in clinical practice. The majority of cases of acute pericarditis in developed countries are caused by viral infections or are autoreactive [6], and bacterial pericarditis is uncommon. Purulent pericarditis is rare, accounting for only < 1% of all cases [7]. Tuberculosis is the most common pathogen in bacterial pericarditis around the world [6], whereas *Staphylococci*, *Streptococci*, and *Pneumococci* are more common in patients with empyema (50%) or pneumonia (33%) respectively [8]. *Salmonella* and *Shigella* are uncommon but important gastrointestinal pathogens associated with myo(peri)carditis; however, the rising incidence of campylobacteriosis worldwide over the last decade has heightened interest in *Campylobacter*-associated myo(peri)carditis [1]. *C. fetus* is almost always isolated from blood in cases where the pathogen has been linked to myopericarditis [9]. In some cases, *C. fetus* was isolated directly from pericardial fluid [3, 4]. *C. coli*-associated myo(peri)carditis has been described in a few case reports [10, 11]. These reports suggested that antecedent *C. coli* enterocolitis, rather than *C. coli* growing from blood or pericardial fluid played a role. We present a case of infectious pericarditis caused by *C. coli* isolated from both pericardial fluid and blood in a patient who did not develop enterocolitis.

## Case presentation

A 72-year-old Japanese man was admitted to the hospital with a fever and a large amount of pericardial fluid. He had been on hemodialysis for the past 19 years due to polycystic kidney disease. He had also been receiving treatment for hypertension and diabetes mellitus for the previous 32 years. His medical history included a urinary infection caused by *Klebsiella pneumoniae*, food poisoning caused by *Salmonella* spp. and oral candidiasis. He had no history of alcohol intake and was not infected with the human immunodeficiency virus.

**Fig. 1:**
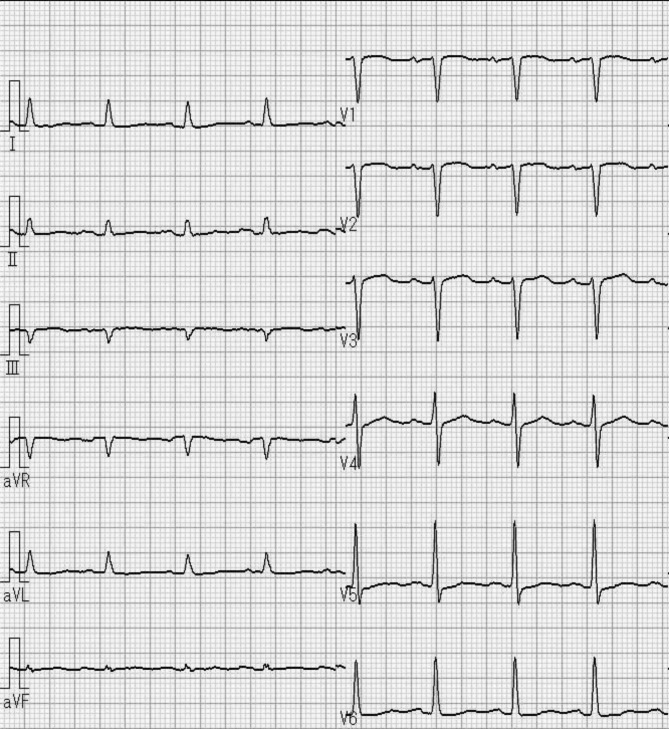
Electrocardiogram three days before admission shows flattening of T wave and poor progression of R wave

He began coughing two weeks before his hospitalization. A chest X-ray and computed tomography (CT) scan one week before hospitalization revealed bilateral pleural fluid and pericardial fluid. An electrocardiogram taken three days before admission revealed flattening of the T wave and poor R wave progression (Fig. 1). Hence, he was admitted to the hospital for further testing.

He was awake but vomited upon admission. There was no chest pain or enteritis in the patient. He had not eaten any raw or undercooked food prior to his admission. His physical exam revealed tachycardia, with a heart rate of 126 beats per minute, blood pressure of 172/84-mm Hg, and an axillary temperature of 38.6 °C. C-reactive protein (39.59 mg/dL [normal range 0.00–0.14]), procalcitonin (49.11 ng/mL [normal range 0–0.5]), hemoglobin A1c (7.8% [normal range 4.9–6.0]), and white blood cell (10,760/µL [normal range 3,300–8,600]) counts were all elevated. Creatinine phosphokinase (35 U/L [normal range 59–248]), and lactate dehydrogenase (238 U/L [normal range 124–222]) were, however, almost normal. Additional laboratory tests revealed that β-D-glucan levels were extremely high (> 300 pg/mL [normal range 0–20]) and aspergillus antigen levels were mildly elevated (0.5 cutoff index [normal range 0–0.4]). Anti-GPL-core IgA antibody, QuantiFERON TB-Gold plus, and cryptococcus antigen were all negative. A transthoracic echocardiogram revealed a significant amount of pericardial fluid. Spiral-shaped, gram-negative rods were cultured from admission blood (two sets) and on day 2 (two sets).

On day 2 of hospitalization, blood-tinged pericardial fluid was drained through pericardiocentesis. The pericardial fluid was examined and discovered to have pyogenic inflammation (41,368/µL leukocytes, 61.0% neutrophils, 2.0% lymphocytes, and 37.0% macrophages) as well as mildly elevated adenosine deaminase (ADA) levels (42.4 U/L). The pericardial pathological examination of the pericardial fluid revealed no bacterium, mycobacterium, fungus, or malignant findings. The spiral-shaped gram-negative rods grew in pericardial fluid culture, similar to the blood culture. Commercial automatic identification systems failed to recognize the organism. The organism was identified as *C. coli* or *C. jejuni* using Matrix-assisted laser desorption ionization time-of-flight analysis, but it could not tell the difference. Finally, using the Hippurate hydrolysis test, the organism was identified as *C. coli*. In the pericardial fluid culture, no mycobacteria or fungi grew.

Drug susceptibility of the organism was evaluated. Minimum inhibitory concentrations against the organism were as follows: ampicillin, 1.0 mg/mL; imipenem, < 1.0 mg/mL; erythromycin, < 2.0 mg/mL; ciprofloxacin, < 0.25 mg/mL; and gentamicin, < 1.0 mg/mL. There stool was not microbiologically examined. Based on the findings, *C. coli* bacteremic pericarditis was diagnosed.

Meropenem (0.5 g, twice daily, renal failure dose) was administered intravenously for 44 days, followed by micafungin (100 mg, once daily) for 15 days. On the sixth day of hospitalization, a transesophageal echocardiogram revealed no evidence of infective endocarditis. Furthermore, no organism grew from the blood on day 6 (two sets). He was discharged from the hospital on day 45 without any sequelae. His treatment was completed 14 days after he was discharged with oral clarithromycin (400 mg, twice daily). Even after 5 months after discharge, the patient never showed signs of disease relapse.

## Discussion and conclusions

Campylobacteriosis has become more common in the last decade, drawing attention to *Campylobacter*-associated myo(peri)carditis [1]. The observed differences in pathogens are notable in our report. In this case, *C. coli* grew from blood and pericardial fluid. In the present case, it was unclear when, and where the patient became infected with *C. col*i in this case. He had no significant incidents, such as animal contact or eating raw meat. He is an elderly immunecompromised patient with diabetes and chronic renal failure on dialysis.

Although the exact mechanism by which *Campylobacter* causes myopericarditis is unknown, several intriguing putative mechanisms [9] have been proposed, including direct microbial invasion via the blood and immunological mechanisms. *C. fetus* pericarditis appears to be the result of pericardium colonization following bacteremia in an immunocompromised host [9]. When the pathogen is linked to myopericarditis, *C. fetus* is almost always isolated from blood [9]. *C. fetus* was isolated from blood in 90% (9/10) cases of *C. fetus* pericarditis. *C. fetus* myopericarditis could be caused by direct microbial invasion of the pericardium via the blood stream. On the other hand, *C. jejuni* causes myo(peri)carditis in immunocompetent hosts [9]. *C. jejuni* was identified in 12 cases of *C. jejuni*-associated myo(peri)carditis through positive stool cultures and/or serological analyses, but it was never isolated from blood [9]. It is unknown whether the cardiac involvement is caused by an immunologic process, as in Guillain–Barré syndrome-related *C. jejun* infection or a direct effect of *Campylobacter* spp. on the myocytes [12]. *C. coli* has properties similar to *C. jejuni* and causes enterocolitis in immunocompetent people. *C. coli*-caused myo(peri)carditis in immunocompetent hosts with enteritis in the reported cases, and *C. coli* was identified from stool [10, 11]. In the present case, *C. coli* caused pericarditis in an immunocompromised host who did not have enteritis, and *C. coli* was isolated from blood and pericardial fluid. Therefore, *C. coli* in the current case may directly invade the pericardium similar to *C. fetus*.

Quinolones and macrolides are frequently used in the treatment of *C. coli* infections [1]. *C. coli* is becoming increasingly resistant to them, according to recent reports [1]. In this case, the antimicrobial susceptibility of the organism was nearly perfect. It is preferable to use narrow-spectrum antibiotics to reduce the possibility of resistance development. However, we are unable to locate the date of antibiotic administration to the pericardial fluid or the clinical outcome. *C. coli* showed elevated minimum inhibitory concentrations to meropenem after long-term oral antibiotic treatment with tebipenem and faropenem for persistent infection, according to a case report [13]. More research combining appropriate treatment is needed.

Laboratory testing revealed a highly elevated β-D-glucan (> 300 pg/mL) and a mildly elevated aspergillus antigen in this case (0.5 cutoff index). When β-D-glucan is high, it generally suggests the presence of fungal infection [14]. When *Candida* or *Aspergillus* fungi were cultured from pericardial fluid, some cases of *Candida* pericarditis or *aspergillus* pericarditis were diagnosed [15–20]. Physical examination, chest CT, and blood and pericardial effusion cultures ruled out fungal pericarditis, fungal pneumonia, and fungal bacteremia. There were no risk factors of β-D-glucan false positives, such as the use of cellulose membrane for dialysis. Despite the fact that β-D-glucan levels dropped to 31.9 pg/mL after 15 days of intravenous micafungin administration, we were unable to detect mycosis throughout the course of the disease.

The pericardial fluid revealed a mildly elevated ADA (42.4 U/L) in the present case. High levels of ADA indicated tuberculous pericarditis. A recent meta-analysis of tuberculous pericarditis reported a pooled sensitivity of 0.90, specificity of 0.85, and negative possibility of 15% [21]. However, it is not a reliable method to distinguish between tuberculous and septic pericarditis. Interferon-gamma release assays (IGRAs) are another test that can detect tuberculous pericarditis. Another recently published meta-analysis of tuberculous pericarditis for IGRA utility discovered a pooled sensitivity of 0.94, specificity of 0.94, and negative likelihood of 6% [22]. IGRA is more sensitive and specific than ADA. Therefore, it is unlikely that the patient in the present case has tuberculous pericarditis.

To summarize, *C. coli*-associated myo(peri)carditis is extremely rare; there have been a few reported cases without proof of positive culture in pericardial effusion. Because *C. coli* was isolated directly from the pericardial fluid in this case, the diagnosis was certain. *C. coli* has been suggested to cause septic pericarditis in immunocompromised hosts, although it typically causes enteritis.

## Data Availability

Not applicable.

## Abbreviations

ADA: Adenosine deaminase
CT: Computed tomography
IGRA: Interferon-gamma release assays
